# Magnetic Field Dependence of Spectral Correlations between ^31^P-Containing Metabolites in Brain

**DOI:** 10.3390/metabo13020211

**Published:** 2023-01-31

**Authors:** Sungtak Hong, Jun Shen

**Affiliations:** Section on Magnetic Resonance Spectroscopy, National Institute of Mental Health, National Institutes of Health, Bethesda, MD 20892, USA

**Keywords:** ^31^P magnetic resonance spectroscopy, magnetic field strengths, linewidth, Pearson’s correlation coefficient, spectral baseline, spectral overlap

## Abstract

Spectral correlations between metabolites in ^31^P magnetic resonance spectroscopy (MRS) spectra of human brain were compared at 3 and 7 Tesla, the two commonly used magnetic field strengths for clinical research. It was found that at both field strengths, there are significant correlations between ^31^P-containing metabolites arising from spectral overlap, and their downfield correlations are markedly altered by the background spectral baseline. Overall, the spectral correlations between ^31^P-containing metabolites are markedly reduced at 7 Tesla with the increased chemical shift dispersion and the decreased membrane phospholipid signal. The findings provide the quantitative landscape of pre-existing correlations in ^31^P MRS spectra due to overlapping signals. Detailed procedures for quantifying the pre-existing correlations between ^31^P-containing metabolites are presented to facilitate incorporation of spectral correlations into statistical modeling in clinical correlation studies.

## 1. Introduction

Phosphorous (^31^P) magnetic resonance spectroscopy (MRS) is an important technique enabling noninvasive assessment of many aspects of bioenergetics and metabolism in vivo [1]. ^31^P MRS has been widely used to study numerous diseases such as Parkinson’s disease [2], tumor [3], diabetes [4], stroke [5], hepatobiliary disease [6], and neuromuscular disorders [7]. In many clinical applications of ^31^P MRS, metabolite signals such as adenosine triphosphate (ATP), phosphocreatine (PCr), inorganic phosphate (P_i_), and phosphoesters are measured in vivo and then correlated with clinical metrics such as disease severity and/or treatment. For example, the duration of illness was shown to be correlated with phosphoesters in patients with Wilson’s disease [8]. The phosphoesters were also found to correlate with the time of transplantation in kidney transplant patients [9]. Phosphocreatine levels in the brain were found to be reduced in cerebral creatine deficiency caused by guanidinoacetate methyltransferase deficiency [10]. After treatment by oral creatine supplementation, brain PCr level was restored [10].

At 3 Tesla or lower magnetic field strengths, there is severe spectral overlap between ATP and nicotinamide adenine dinucleotide (NAD) and among the signals downfield from PCr, which are phosphocholine (PC), glycerophosphocholine (GPC), intra- and extracellular inorganic phosphate (P_i_^in^, P_i_^ex^), phosphoethanolamine (PE), and glycerophosphoethanolamine (GPE) [11]. In particular, the signals from macromolecule membrane phospholipids (MP) are highly prominent at low magnetic fields [12], overlapping with the downfield signals. At the high magnetic field strength of 7 Tesla, spectral overlap between ^31^P-containing metabolites is greatly reduced, as it is aided by the increased chemical shift dispersion and the large decrease in the signal intensity of MP. However, even at 7 Tesla, there is still severe spectral overlap among ATP, NAD, and uridine diphosphate glucose (UDPG) [11].

In the clinical ^31^P MRS literature, concentrations of ^31^P-contaning metabolites measured by ^31^P MRS have been treated as statistically independent variables in their correlations with clinical metrics. However, spectral overlap can present as an intervening variable that may unfortunately reduce the likelihood of the intended statistical precision and extrapolation of the clinical findings under investigation, unless correlations of metabolites can be parsed correctly [13,14]. When two peaks of the same polarity overlap each other, overdetermination (underdetermination) of one peak is correlated with the underdetermination (overdetermination) of the other, leading to a negative correlation between the two signals [13]. To the best of our knowledge, correlations originating from spectral overlap have not been taken into account in clinical MRS studies, including clinical studies using ^31^P MRS, although these spectral correlations may confound correlations of biological origins.

Monte Carlo analysis has been a standard tool to quantify correlations [13]. In this study, we use numerical Monte Carlo simulations to investigate and quantify spectral correlations between ^31^P-containing brain metabolites in the absence of any influence from biological correlations. To evaluate the effect of magnetic field strength on these spectral correlations, in vivo ^31^P MRS data measured at both 3 and 7 Tesla were analyzed. Highly significant spectral correlations were found between the oxidized and reduced forms of NAD at both 3 and 7 Tesla. Significant correlations were also found between many downfield ^31^P MRS signals, especially at 3 Tesla. In addition, although the background spectral baseline has little effect on correlations between the upfield signals, it plays a major role in downfield correlations. The complexity of the spectral correlations between ^31^P-containing metabolites underscores the importance of quantifying these correlations and incorporating them in statistical modeling of the correlations between overlapping ^31^P-containing metabolites and clinical metrics.

## 2. Materials and Methods

### 2.1. In Vivo ^31^P MRS Data

Ten in vivo ^31^P MRS datasets, which were acquired from five healthy participants (mean age 26.8 ± standard deviation 7.7 years) at 3 Tesla and five healthy participants (age 33.5 ± 10.3 years) at 7 Tesla [11], were analyzed in this study. The data acquisition procedures have been described previously [11]. Briefly, MRS measurements were performed on Siemens Skyra 3 Tesla and Magnetom 7 Tesla scanners (Siemens Healthcare, Erlangen, Germany) using home-built coil assemblies composed of a circular ^31^P coil with a 7.0 cm diameter and a quadrature half-volume ^1^H coil. In vivo ^31^P MRS spectra were acquired without ^1^H decoupling. Acquisition parameters at 3 Tesla were as follows: TR = 2 s; spectral width = 5 kHz; number of acquisitions = 128; number of data points = 1024. Identical parameters were used for 7 Tesla data acquisition, except that TR = 3 s.

### 2.2. In Vivo Data Processing and Quantification

Identical data processing procedures were used for MRS data acquired at both field strengths. The first two data points in FID were set to zero to suppress the baseline in the ^31^P MRS spectrum [15]. Subsequently, zero-filling, 1-Hz exponential line-broadening, Fourier transform, zero- and first-order phase corrections, and chemical shift referencing (by setting PCr to 0 ppm) were performed.

All spectra were quantified using an in-house-developed fitting program [16] implementing the linear combination model (LCM)-fitting algorithm [17]. Basis data, a prerequisite for LCM fitting, were generated with density matrix simulations [18]. The following 12 metabolites covering the spectral region between −20 to 10 ppm were included in the spectral model: PCr, ATP, the oxidized form of NAD (NAD^+^), the reduced form of NAD (NADH), UDPG, GPC, GPE, P_i_^ex^, P_i_^in^, PC, PE, and MP. Both chemical shifts and coupling constants, which must be priorly known for density matrix simulation, were taken from the literature [19,20]. Due to the low sensitivity at 3 Tesla, UDPG was included only in the basis data of 7 Tesla. During the spectral fitting, the linewidths of NAD^+^ and NADH were constrained to the linewidth of α-ATP minus 1.5 Hz [21]. The background spectral baseline was modeled using a polynomial. The whole spectral region which covered −20 to 10 ppm was quantified at both field strengths. For calculating metabolite concentrations, total ATP (the sum of α-, β-, and γ-ATP) was used as an internal reference, and it was assumed to be 9 mM [22].

### 2.3. Monte Carlo Analysis of In Vivo Spectra

The first Monte Carlo analysis was performed to investigate the effects of field strengths and the background spectral baseline on metabolite–metabolite correlations. For each participant, two different datasets composed of metabolites only and metabolite + baseline were generated using fitted individual metabolites and the background spectral baseline derived from LCM fitting. Subsequently, random noise at the same level derived from the corresponding in vivo spectrum was added. A separate dataset excluding the spectral baseline was also generated using the same procedure to investigate the influence of the background spectral baseline on metabolite–metabolite correlations. For each dataset, 2000 different noise realizations were used, resulting in a total of 40,000 spectra (=2 field strengths × 5 participants × 2 baseline options × 2000 noise realizations).

The second Monte Carlo analysis was performed to investigate the effects of linewidth on metabolite–metabolite correlations at different field strengths. The mean linewidth and amplitude of each metabolite and the mean spectral baseline extracted from the LCM fitting of all participants were used to generate a noiseless synthetic spectrum for each field strength. For 3 Tesla, three different line-broadening factors of 0, 4, and 8 Hz were used to study the effect of linewidth variations. For 7 Tesla, the line-broadening factors were 0, 10, and 20 Hz. A separate dataset that excluded the spectral baseline was also generated for each field strength using the same procedure to investigate the influence of the spectral baseline on metabolite–metabolite correlations. For each dataset, random noise with 2000 different noise realizations at the corresponding mean in vivo noise level was added to each synthetic spectrum. A total of 24,000 spectra (=2 field strengths × 3 different line-broadening factors × 2 baseline options × 2000 noise realizations) were generated. All 24,000 spectra were quantified using the same LCM fitting procedure as described in Section 2.2.

### 2.4. Correlation Analysis

Pearson’s cross-correlation coefficients between pairs of ^31^P-containing metabolites + MP were calculated using the fitted concentrations of individual signals derived from Monte Carlo analysis. Then, the Pearson’s cross-correlation coefficients were used to investigate the influence of spectral baseline and linewidths at different field strengths.

All computational tasks, including density matrix simulation, data processing, Monte-Carlo analysis, and correlation analysis, were carried out with in-house software written in MATLAB (R2020b; MathWorks, Natick, MA, USA) on a personal laptop using an Intel CoreI i7-10850H CPU (2.7 GHz) with 32 GB RAM.

## 3. Results

**Figure 1:**
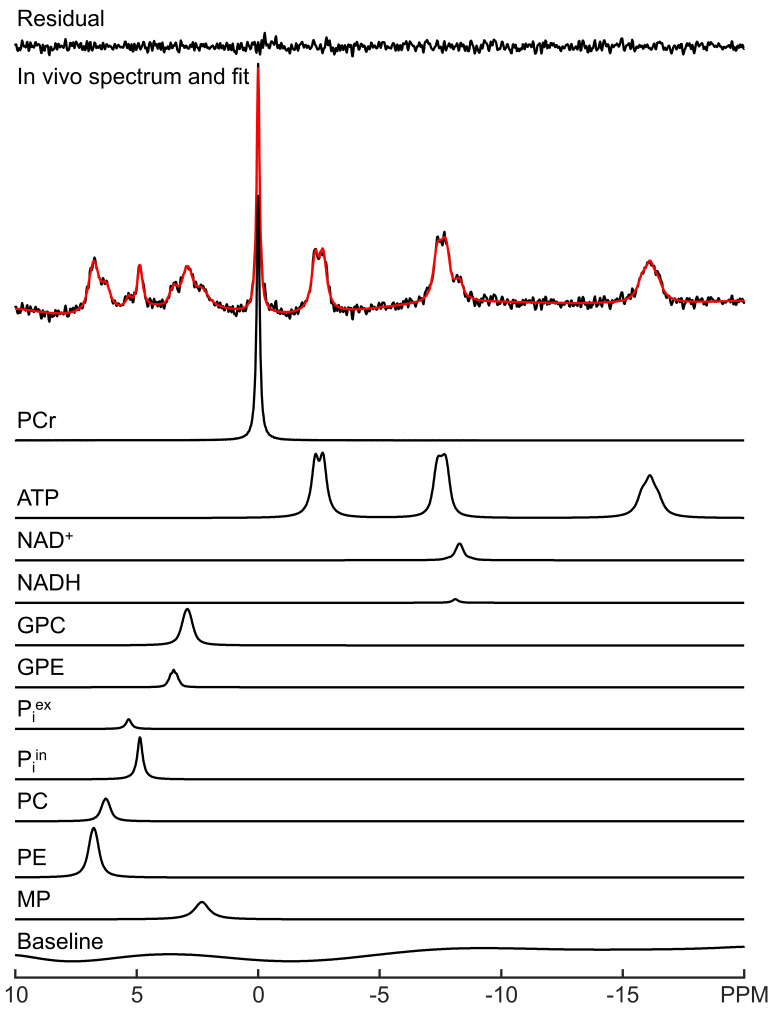
LCM fitting of a representative in vivo ^31^P MRS spectrum acquired from human brain at 3 Tesla. The fit (red) and fit residual covering −20 to 10 ppm are shown, along with fitted individual metabolites and the background spectral baseline.

**Figure 2:**
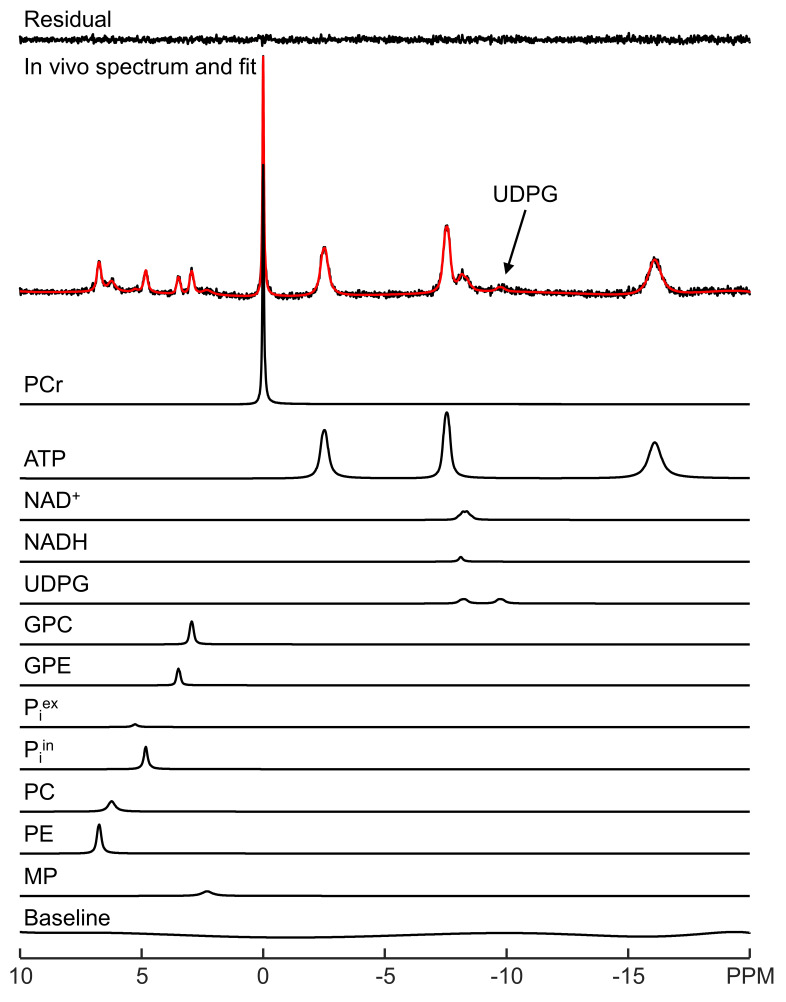
LCM fitting of a representative in vivo ^31^P MRS spectrum acquired from human brain at 7 Tesla. The fit (red) and fit residual covering −20 to 10 ppm are shown, along with fitted individual metabolites and the background spectral baseline.

LCM spectral fitting of a representative in vivo ^31^P MRS spectrum acquired from human brain at 3 Tesla is shown in Figure 1. The fitting result for a representative 7 Tesla spectrum is shown in Figure 2. Note that although UDPG was not detected in individual participants at 3 Tesla, the improved sensitivity at 7 Tesla allows the detection of UDPG indicated by an arrow in Figure 2. At 7 Tesla, the MP signal is greatly reduced, a reduction which is accompanied by substantially improved spectral resolution in the downfield region. The background spectral baseline is also significantly reduced at 7 Tesla. The PCr linewidth and SNR were found to be 6.3 ± 0.9 Hz and 82 ± 21 at 3 Tesla and 9.3 ± 2.1 Hz and 171 ± 27 at 7 Tesla, respectively. The means and standard deviations of metabolite concentrations found by LCM fitting are summarized in Supplementary Table S1. The results are in agreement with a previous study that used jMRUI [11].

**Figure 3:**
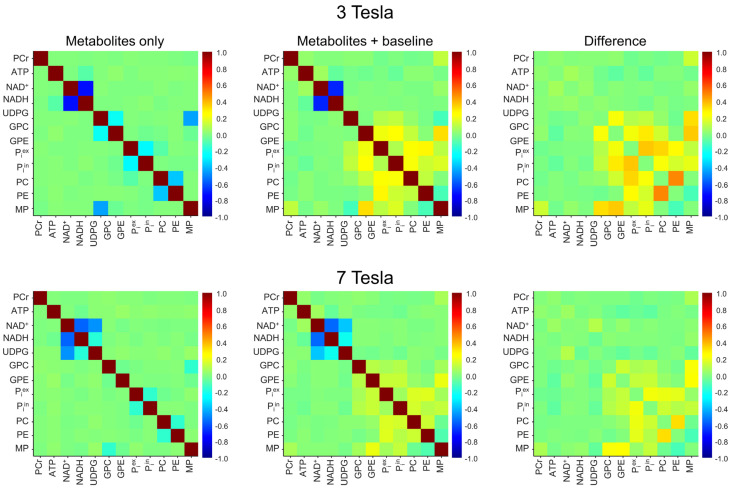
Mean cross-correlation coefficient matrices derived from Monte Carlo analysis of the in vivo 3 Tesla (n = 5; top row) and 7 Tesla (n = 5; bottom row) ^31^P MRS spectra. The Monte Carlo analysis was performed for two different scenarios: metabolites only (first column) and metabolites + baseline (second column). The matrices in the third column represent the differences obtained by subtracting the matrices in the first column from those in the second column.

The 3 Tesla spectral model contains 11 × 10/2 = 55 pairs of ^31^P-containing metabolites + MP. From the Monte Carlo analysis of the 10,000 spectra without the background spectral baseline, Pearson’s correlation coefficients for each of the 55 pairs were computed. The results were depicted by the top-left correlation coefficient matrix in Figure 3. As seen in this matrix, there is a strong negative correlation between NAD^+^ and NADH. Without the spectral baseline, the correlation coefficient of the NAD^+^–NADH at 3 Tesla was found to be −0.72 ± 0.04 (n = 5). Except for NAD^+^–NADH, GPC–GPE, GPC–MP, P_i_^ex^–P_i_^in^, and PC–PE, all other metabolite–metabolite pairs showed only minor correlations with Pearson’s correlation coefficients in the range of −0.1~0.1.

The effect of the background baseline at 3 Tesla is shown by the middle and the right matrices in the top row of Figure 3. These correlation coefficients were computed from the 10,000 spectra that include the background spectral baseline. As seen in these two matrices, the addition of the background spectral baseline has little effect on the upfield correlations at 3 Tesla. For example, the NAD^+^–NADH correlation coefficient was found to be −0.68 ± 0.05 (n = 5) with the background spectral baseline, which is comparable to the corresponding value in the absence of the background spectral baseline (−0.72 ± 0.04, n = 5; effect size = 0.94, *p* = 0.008). However, the background spectral baseline causes relatively large changes in the downfield correlations, as evidenced by the top right difference matrix.

The procedure for computing the correlation coefficients at 7 Tesla is similar. The results are shown by matrices in the bottom row of Figure 3. As expected, all correlations become weaker at 7 Tesla due to the increased spectral resolution. Note that the 7 Tesla spectral model includes UDPG; therefore, it contains 12 × 11/2 = 66 pairs of ^31^P-containing metabolites + MP. The pair with the greatest correlation coefficient at 7 Tesla remains NAD^+^–NADH. However, the magnitude of their correlation becomes smaller than at 3 Tesla. Without the background spectral baseline, the NAD^+^–NADH correlation coefficient was found to be −0.56 ± 0.01 (n = 5) at 7 Tesla. With the background spectral baseline, this correlation is barely changed (−0.55 ± 0.01; n = 5; effect size = 0.73, *p* = 0.14). Similar to the 3 Tesla case, the background spectral baseline causes relatively large changes in the downfield correlations, although the overall change is much smaller than at 3 Tesla.

**Figure 4:**
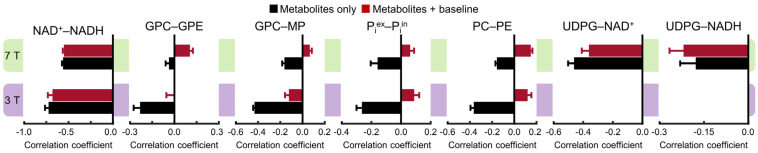
Mean cross-correlation coefficients for representative pairs of metabolites derived from Monte Carlo analysis of the 3 Tesla (n = 5) and 7 Tesla (n = 5) ^31^P MRS spectra. Monte Carlo analysis was performed for two different scenarios: metabolites only (black) and metabolites + baseline (dark red). Error bars represent standard deviations.

A more detailed comparison is shown in Figure 4, which demonstrates the complex effect of the background spectral baseline at both 3 and 7 Tesla. Notably, there are highly significant correlations between UDPG and NAD (both NAD^+^ and NADH) at 7 Tesla. Although the background spectral baseline has a relatively small effect on the UDPG–NAD correlations, the influence of the baseline is quite significant for the downfield correlations, especially at 3 Tesla. For example, the PC–PE correlations at 3 Tesla changed from −0.36 ± 0.03 (n = 5) without the baseline to 0.12 ± 0.03 (n = 5; effect size = 14.99, *p* < 0.0005) with the baseline.

**Figure 5:**
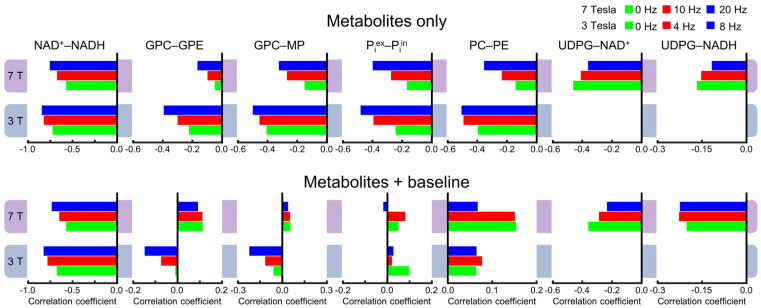
Mean cross-correlation coefficients with metabolites only (top row) and with metabolites + baseline (bottom row) derived from Monte Carlo analysis. Three different line-broadening factors of 0, 4, and 8 Hz for 3 Tesla and 0, 10, and 20 Hz for 7 Tesla were applied.

Because spectral overlap becomes more prominent with broader lines, line-broadening is expected to increase cross-correlations in general. As described in the Materials and Methods, 4000 spectra were generated for each line-broadening factor at each field strength, with half of the spectra without the background spectral baseline. Figure 5 shows examples of the cross-correlation coefficients with different line-broadening factors. The numerical values of all correlation coefficients for characterizing the linewidth effect are provided in Supplementary Tables S2 and S3. As shown in Figure 5, there is a trend by which the magnitude of the cross-correlation coefficients becomes greater with broader linewidths at both field strengths. However, there are exceptions to this trend. For example, the Pearson’s correlations coefficients for the UDPG–NAD^+^ pair become smaller with greater line-broadening with and without the background spectral baseline over the linewidth range investigated here. Also shown by Figure 5, adding the background spectral baseline has a strong impact on cross-correlation coefficients for metabolite pairs such as GPC–GPE, GPC–MP, P_i_^ex^–P_i_^in^, and PC–PE, which belong to the downfield spectral region. This strong impact disrupts the trend of shared directionality of correlation and linewidth.

A secondary effect of line-broadening is degraded measurement precision. Table 1 and Table 2 summarize the linewidth effect on coefficients of variation (CVs) of the ^31^P-containing metabolites at both 3 and 7 Tesla. As expected, the results demonstrate increased CVs when the linewidths are increased. Adding the background spectral baseline also increases CVs as the baseline contributes to the overall crowdedness.

## 4. Discussion

In this study, we have shown that many ^31^P-containing metabolites measured using in vivo ^31^P MRS are correlated due to spectral overlap. Like in the case of short echo time proton MRS these correlations are influenced by linewidth and interactions with the background spectral baseline [23,24], especially in the downfield region. Compared with 3 Tesla, metabolite–metabolite correlations are significantly reduced at 7 Tesla, highlighting a previously underappreciated benefit of high magnetic field MRS. Even at 7 Tesla, however, there remain significant metabolite–metabolite correlations due to the severe spectral overlap between NAD^+^, NADH, and UDPG, and, to a lesser extent, due to interactions with the background spectral baseline in the downfield region.

Correlation between two variables A and B can be distorted by their correlation with a potentially intervening variable C. The influence of variable C can be illustrated using partial correlations. Specifically, the partial correlation coefficient between A and B with the influence of C excluded (r_AB|C_) is defined as follows [24,25]:

(1) $$r_{AB|C}=\frac{r_{AB}-r_{AC}r_{BC}}{\sqrt{1-r_{AC}^{2}}\sqrt{1-r_{BC}^{2}}}$$

where r_AB_, r_AC,_ and r_BC_ are Pearson’s correlation coefficients measured experimentally. In the case of correlating a ^31^P-containing metabolite (A) with a clinical metric (B), the measured Pearson’s correlation coefficient r_AB_ could be significantly influenced by another ^31^P MRS signal (C) overlapping with the ^31^P signal of interest (A). It is well known in statistics that if the r_AB|C_ is much smaller than r_AB_, then the Pearson’s correlation between A and B is considered spurious, which would be caused by their correlations with C instead of by the genuine correlation between A and B [25,26].

In proton MRS, it is often possible to suppress the influence of overlapping signals by spectral editing or by altering the echo time [27]. Unfortunately, ^31^P signals generally have very short T_2_; therefore, it is difficult to spectroscopically suppress spectral overlap in ^31^P MRS. These considerations and the results of this study indicate that metabolite–metabolite correlations due to spectral overlap need to be considered in downstream statistical correlations between overlapping ^31^P-containing metabolites and clinical metrics. Furthermore, compared to the 3 Tesla results, the dramatic reduction in metabolite–metabolite correlations at 7 Tesla highlights the advantage of high magnetic field ^31^P MRS in avoiding or reducing the confounding spectral correlations.

ATP comprises three moieties that resonate at −7.56 ppm (α-ATP), −16.18 ppm (β-ATP), and −2.53 ppm (γ-ATP). Of them, the α-ATP peak overlaps strongly with both NAD^+^ and NADH. In contrast, β-ATP and γ-ATP are not affected by any spectral overlap. As such, ATP as a whole is well determined, even in the presence of significant spectral overlap between α-ATP and NAD. This de facto spectral separation between ATP and NAD is reflected by the negligible Pearson’s correlation coefficients for ATP–NAD^+^ and ATP–NADH, even at 3 Tesla.

It is well-known in the MRS literature that line-broadening due to poor shimming degrades measurement precision. This effect of line-broadening is reflected by the results of Table 1 and Table 2, which show increased metabolite CVs due to increased line-broadening with and without the background spectral baseline and at both field strengths. Note that the increased correlation between overlapping signals due to line-broadening also contributes to increased CVs because of the greater measurement uncertainty in the presence of spectral overlap. In contrast to the dependence of CVs on linewidth, the effects of line-broadening on metabolite–metabolite correlation are more nuanced. Although the general trend of correlation increasing with broader resonance lines is expected, it is noted that metabolite–metabolite correlations are also influenced by other overlapping resonances in a complex and often nonintuitive fashion. Equation (1) above provides an illustrating example for the simple case of three-way correlations. Therefore, it is not surprising that, over the range of 20 Hz, it was found that line-broadening reduces Pearson’s correlation between UDPG–NAD^+^, as shown in Figure 5.

## 5. Conclusions

Using Monte Carlo analysis with the exclusion of biological effects, the metabolite–metabolite correlations due to spectral overlap in the ^31^P MRS of human brain were systematically investigated. The results show the importance of high magnetic fields in reducing the confounding metabolite–metabolite correlations due to overlapping ^31^P MRS signals. The quantification of the pre-existing correlations between ^31^P-containing metabolites described in this work is expected to facilitate clinical studies that involve correlating overlapping ^31^P MRS signals with clinical metrics.

## Figures and Tables

**Table 1:** Effect of line-broadening on coefficient of variation (CV) at 3 Tesla.

	PCr	ATP	NAD^+^	NADH	GPC	GPE	P_i_^ex^	P_i_^in^	PC	PE	MP
Metabolites only											
CV (0 Hz)	0.65	0.67	9.30	37.20	2.40	4.51	9.22	2.82	4.27	1.83	2.91
CV (10 Hz)	0.79	0.74	12.46	53.00	2.74	5.36	10.81	3.47	4.80	2.11	3.19
CV (20 Hz)	0.93	0.75	15.27	66.54	3.19	5.95	12.96	4.04	5.55	2.31	3.51
Metabolites + baseline											
CV (0 Hz)	0.70	0.85	9.76	37.24	2.53	5.34	11.17	3.28	5.20	2.31	4.17
CV (10 Hz)	0.91	0.97	13.21	51.58	2.92	6.55	13.50	4.08	6.16	2.65	4.68
CV (20 Hz)	1.05	1.05	16.84	67.77	3.46	7.69	15.41	4.92	6.85	3.05	5.20

**Table 2:** Effect of line-broadening on coefficient of variation (CV) at 7 Tesla.

	PCr	ATP	NAD^+^	NADH	UDPG	GPC	GPE	P_i_^ex^	P_i_^in^	PC	PE	MP
Metabolites only												
CV (0 Hz)	0.31	0.37	5.30	14.55	7.31	1.99	2.76	10.40	1.99	3.35	1.42	7.04
CV (10 Hz)	0.45	0.40	6.48	20.44	8.08	2.44	3.50	12.23	2.47	4.05	1.68	7.53
CV (20 Hz)	0.56	0.44	8.21	27.52	8.84	3.00	4.11	14.96	2.97	4.75	1.97	8.22
Metabolites + baseline												
CV (0 Hz)	0.34	0.44	5.32	15.08	8.15	2.06	3.05	11.82	2.19	4.05	1.65	9.00
CV (10 Hz)	0.50	0.51	6.58	20.18	9.71	2.68	4.12	14.95	2.89	4.99	2.04	9.97
CV (20 Hz)	0.61	0.57	8.12	27.31	11.11	3.28	4.97	17.56	3.59	5.81	2.37	11.34

## Data Availability

The datasets are available from the corresponding author on request.

## Supplementary Materials

The following supporting information can be downloaded at: https://www.mdpi.com/article/10.3390/metabo13020211/s1, Table S1: Means and standard deviations of metabolite concentrations obtained at 3 Tesla (n = 5; upper row) and 7 Tesla (n = 5; lower row); Table S2: Mean cross-correlation coefficients derived from Monte Carlo analysis performed with metabolites only (left panel) and metabolites + baseline (right panel) using three different line-broadening factors of 0 Hz (top row), 4 Hz (middle row), and 8 Hz (bottom row) at 3 Tesla; Table S3: Mean cross-correlation coefficients derived from Monte Carlo analysis performed with metabolites only (left panel) and metabolites + baseline (right panel) using three different line-broadening factors of 0 Hz (top row), 10 Hz (middle row), and 20 Hz (bottom row) at 7 Tesla.
