# Supplementary material for: Magnetic Field Dependence of Spectral Correlations between 31P-Containing Metabolites in Brain
Source: Metabolites. 2023 Jan 31;13(2):211. doi: 10.3390/metabo13020211 (PMC9967573; doi:10.3390/metabo13020211)
Supplement: Supplementary file 1 [file metabolites-13-00211-s001.zip › metabolites-2172632-supplementary.pdf]

Supplementary Materials

Supplementary Table S1. Means and standard deviations of metabolite concentrations obtained at 3 Tesla (n = 5; upper row) and 7 Tesla (n = 5; lower row). All values were expressed in mM.

		PCr	α-ATP	β-ATP	γ-ATP	NAD^+	NADH	UDPG	GPC	GPE	P_i^{ex}	P_i^{in}	PC	PE	MP
3 Tesla*	Mean	2.92	2.99	2.68	3.33	0.220	0.048	N/A	1.41	0.60	0.30	0.88	0.74	1.78	1.69
	SD	0.31	0.11	0.06	0.13	0.031	0.005	N/A	0.15	0.11	0.07	0.09	0.17	0.23	0.37
7 Tesla	Mean	2.79	3.03	3.23	2.74	0.275	0.071	0.231	0.61	0.42	0.15	0.66	0.44	1.02	0.36
	SD	0.32	0.10	0.23	0.15	0.010	0.017	0.071	0.11	0.10	0.11	0.05	0.05	0.11	0.12

*See Reference [11] for quantification of the downfield metabolites.

Supplementary Table S2. Mean cross-correlation coefficients derived from Monte Carlo analysis performed with metabolites only (left panel) and metabolites + baseline (right panel) using three different line-broadening factors of 0 Hz (top row), 4 Hz (middle row), and 8 Hz (bottom row) at 3 Tesla.

Metabolite only (LW = 0 Hz)

	PCr	ATP	NAD^+	NADH	GPC	GPE	P_i^{ex}	P_i^{in}	PC	PE	MP
PCr	1.00	0.00	0.00	-0.01	0.05	-0.01	0.00	-0.03	0.00	0.03	-0.03
ATP	0.00	1.00	0.06	-0.15	-0.01	-0.01	-0.01	0.04	0.02	0.01	0.01
NAD^+	0.00	0.06	1.00	-0.72	0.00	0.03	0.02	0.01	0.05	0.00	0.00
NADH	-0.01	-0.15	-0.72	1.00	-0.01	-0.03	-0.02	-0.01	-0.03	0.00	0.00
GPC	0.05	-0.01	0.00	-0.01	1.00	-0.22	-0.02	0.00	-0.01	0.02	-0.41
GPE	-0.01	-0.01	0.03	-0.03	-0.22	1.00	-0.01	-0.04	-0.03	0.02	-0.09
P_i^{ex}	0.00	-0.01	0.02	-0.02	-0.02	-0.01	1.00	-0.24	-0.09	0.01	0.02
P_i^{in}	-0.03	0.04	0.01	-0.01	0.00	-0.04	-0.24	1.00	-0.02	0.01	-0.05
PC	0.00	0.02	0.05	-0.03	-0.01	-0.03	-0.09	-0.02	1.00	-0.40	-0.01
PE	0.03	0.01	0.00	0.00	0.02	0.02	0.01	0.01	-0.40	1.00	0.00
MP	-0.03	0.01	0.00	0.00	-0.41	-0.09	0.02	-0.05	-0.01	0.00	1.00

Metabolite + baseline (LW = 0 Hz)

	PCr	ATP	NAD^+	NADH	GPC	GPE	P_i^{ex}	P_i^{in}	PC	PE	MP
PCr	1.00	0.07	0.00	-0.01	0.06	0.02	-0.01	-0.03	-0.02	0.01	0.12
ATP	0.07	1.00	0.08	-0.10	-0.07	-0.06	-0.04	-0.03	-0.03	0.03	-0.02
NAD^+	0.00	0.08	1.00	-0.68	0.01	0.04	0.00	0.01	0.01	-0.01	0.03
NADH	-0.01	-0.10	-0.68	1.00	-0.01	-0.03	-0.03	-0.02	-0.04	-0.01	0.00
GPC	0.06	-0.07	0.01	-0.01	1.00	-0.01	0.07	0.13	0.01	-0.03	-0.06
GPE	0.02	-0.06	0.04	-0.03	-0.01	1.00	0.21	0.22	0.08	0.01	0.28
P_i^{ex}	-0.01	-0.04	0.00	-0.03	0.07	0.21	1.00	0.10	0.23	0.24	0.14
P_i^{in}	-0.03	-0.03	0.01	-0.02	0.13	0.22	0.10	1.00	0.19	0.13	0.18
PC	-0.02	-0.03	0.01	-0.04	0.01	0.08	0.23	0.19	1.00	0.06	-0.02
PE	0.01	0.03	-0.01	-0.01	-0.03	0.01	0.24	0.13	0.06	1.00	-0.12
MP	0.12	-0.02	0.03	0.00	-0.06	0.28	0.14	0.18	-0.02	-0.12	1.00

Metabolite only (LW = 4 Hz)

	PCr	ATP	NAD^+	NADH	GPC	GPE	P_i^{ex}	P_i^{in}	PC	PE	MP
PCr	1.00	0.02	0.01	-0.02	0.05	0.01	0.03	0.03	0.01	0.02	-0.03
ATP	0.02	1.00	0.05	-0.14	0.01	-0.01	0.01	0.01	0.01	0.01	0.02
NAD^+	0.01	0.05	1.00	-0.82	0.01	-0.03	0.01	-0.02	0.00	0.04	0.02
NADH	-0.02	-0.14	-0.82	1.00	-0.01	0.02	-0.01	0.04	0.01	-0.02	-0.02
GPC	0.05	0.01	0.01	-0.01	1.00	-0.30	0.01	0.00	0.00	0.01	-0.45
GPE	0.01	-0.01	-0.03	0.02	-0.30	1.00	-0.01	-0.03	0.02	-0.01	-0.06
P_i^{ex}	0.03	0.01	0.01	-0.01	0.01	-0.01	1.00	-0.39	-0.13	0.01	-0.05
P_i^{in}	0.03	0.01	-0.02	0.04	0.00	-0.03	-0.39	1.00	0.04	-0.01	-0.01
PC	0.01	0.01	0.00	0.01	0.00	0.02	-0.13	0.04	1.00	-0.49	0.06
PE	0.02	0.01	0.04	-0.02	0.01	-0.01	0.01	-0.01	-0.49	1.00	-0.01
MP	-0.03	0.02	0.02	-0.02	-0.45	-0.06	-0.05	-0.01	0.06	-0.01	1.00

Metabolite + baseline (LW = 4 Hz)

	PCr	ATP	NAD^+	NADH	GPC	GPE	P_i^{ex}	P_i^{in}	PC	PE	MP
PCr	1.00	0.08	-0.05	0.03	-0.02	0.06	-0.04	-0.02	-0.04	-0.01	0.16
ATP	0.08	1.00	0.03	-0.05	-0.02	-0.05	-0.03	-0.01	-0.02	0.05	-0.05
NAD^+	-0.05	0.03	1.00	-0.78	0.03	-0.01	0.02	0.03	0.04	-0.01	-0.01
NADH	0.03	-0.05	-0.78	1.00	-0.02	0.03	-0.03	-0.02	-0.05	-0.01	0.03
GPC	-0.02	-0.02	0.03	-0.02	1.00	-0.07	0.07	0.14	0.08	0.01	-0.12
GPE	0.06	-0.05	-0.01	0.03	-0.07	1.00	0.23	0.28	0.13	-0.01	0.35
P_i^{ex}	-0.04	-0.03	0.02	-0.03	0.07	0.23	1.00	0.02	0.24	0.28	0.12
P_i^{in}	-0.02	-0.01	0.03	-0.02	0.14	0.28	0.02	1.00	0.29	0.12	0.21
PC	-0.04	-0.02	0.04	-0.05	0.08	0.13	0.24	0.29	1.00	0.08	-0.02
PE	-0.01	0.05	-0.01	-0.01	0.01	-0.01	0.28	0.12	0.08	1.00	-0.13
MP	0.16	-0.05	-0.01	0.03	-0.12	0.35	0.12	0.21	-0.02	-0.13	1.00

Metabolite only (LW = 8 Hz)

	PCr	ATP	NAD^+	NADH	GPC	GPE	P_i^{ex}	P_i^{in}	PC	PE	MP
PCr	1.00	-0.03	0.02	0.00	0.02	0.00	-0.01	-0.04	0.00	0.00	-0.08
ATP	-0.03	1.00	0.01	-0.13	0.04	0.00	0.03	-0.01	0.02	0.02	-0.01
NAD^+	0.02	0.01	1.00	-0.85	0.00	-0.01	-0.06	0.03	0.04	-0.04	-0.02
NADH	0.00	-0.13	-0.85	1.00	0.01	-0.02	0.04	-0.02	-0.05	0.04	0.03
GPC	0.02	0.04	0.00	0.01	1.00	-0.39	-0.01	0.01	0.03	-0.02	-0.50
GPE	0.00	0.00	-0.01	-0.02	-0.39	1.00	0.01	-0.05	0.02	0.00	-0.01
P_i^{ex}	-0.01	0.03	-0.06	0.04	-0.01	0.01	1.00	-0.48	-0.21	0.05	0.02
P_i^{in}	-0.04	-0.01	0.03	-0.02	0.01	-0.05	-0.48	1.00	0.02	0.02	-0.04
PC	0.00	0.02	0.04	-0.05	0.03	0.02	-0.21	0.02	1.00	-0.51	-0.03
PE	0.00	0.02	-0.04	0.04	-0.02	0.00	0.05	0.02	-0.51	1.00	0.02
MP	-0.08	-0.01	-0.02	0.03	-0.50	-0.01	0.02	-0.04	-0.03	0.02	1.00

Metabolite + baseline (LW = 8 Hz)

	PCr	ATP	NAD^+	NADH	GPC	GPE	P_i^{ex}	P_i^{in}	PC	PE	MP
PCr	1.00	0.05	0.01	0.02	-0.02	0.03	-0.07	-0.03	-0.03	-0.07	0.15
ATP	0.05	1.00	0.06	-0.10	-0.04	-0.04	-0.02	-0.04	0.01	0.02	0.00
NAD^+	0.01	0.06	1.00	-0.82	-0.02	0.02	-0.04	-0.03	-0.02	-0.03	0.06
NADH	0.02	-0.10	-0.82	1.00	0.02	-0.02	0.03	0.03	0.03	0.03	-0.04
GPC	-0.02	-0.04	-0.02	0.02	1.00	-0.15	0.13	0.13	0.06	0.01	-0.22
GPE	0.03	-0.04	0.02	-0.02	-0.15	1.00	0.25	0.31	0.17	0.03	0.39
P_i^{ex}	-0.07	-0.02	-0.04	0.03	0.13	0.25	1.00	0.03	0.22	0.31	0.08
P_i^{in}	-0.03	-0.04	-0.03	0.03	0.13	0.31	0.03	1.00	0.28	0.15	0.25
PC	-0.03	0.01	-0.02	0.03	0.06	0.17	0.22	0.28	1.00	0.06	-0.01
PE	-0.07	0.02	-0.03	0.03	0.01	0.03	0.31	0.15	0.06	1.00	-0.12
MP	0.15	0.00	0.06	-0.04	-0.22	0.39	0.08	0.25	-0.01	-0.12	1.00

Supplementary Table S3. Mean cross-correlation coefficients derived from Monte Carlo analysis performed with metabolites only (left panel) and metabolites + baseline (right panel) using three different line-broadening factors of 0 Hz (top row), 10 Hz (middle row), and 20 Hz (bottom row) at 7 Tesla.

Metabolites only (LW = 0 Hz)

	PCr	ATP	NAD$^+$	NADH	UDPG	GPC	GPE	P_i^{ex}	P_i^{in}	PC	PE	MP
PCr	1.00	-0.03	0.01	-0.01	0.01	0.02	0.02	0.01	-0.02	0.03	-0.03	-0.01
ATP	-0.03	1.00	0.01	-0.03	0.01	0.02	0.04	0.02	0.02	0.03	0.02	0.04
NAD$^+$	0.01	0.01	1.00	-0.57	-0.46	0.00	-0.03	0.02	-0.02	-0.02	0.01	0.03
NADH	-0.01	-0.03	-0.57	1.00	-0.17	0.00	0.05	-0.02	0.05	0.02	0.04	-0.04
UDPG	0.01	0.01	-0.46	-0.17	1.00	0.00	0.01	0.00	0.00	0.02	-0.02	0.04
GPC	0.02	0.02	0.00	0.00	0.00	1.00	-0.05	0.00	0.02	0.01	-0.02	-0.15
GPE	0.02	0.04	-0.03	0.05	0.01	-0.05	1.00	-0.01	-0.03	0.01	0.03	-0.05
P_i^{ex}	0.01	0.02	0.02	-0.02	0.00	0.00	-0.01	1.00	-0.17	-0.03	-0.01	0.02
P_i^{in}	-0.02	0.02	-0.02	0.05	0.00	0.02	-0.03	-0.17	1.00	0.00	0.00	-0.01
PC	0.03	0.03	-0.02	0.02	0.02	0.01	0.01	-0.03	0.00	1.00	-0.14	0.00
PE	-0.03	0.02	0.01	0.04	-0.02	-0.02	0.03	-0.01	0.00	-0.14	1.00	0.03
MP	-0.01	0.04	0.03	-0.04	0.04	-0.15	-0.05	0.02	-0.01	0.00	0.03	1.00

Metabolites + baseline (LW = 0 Hz)

	PCr	ATP	NAD$^+$	NADH	UDPG	GPC	GPE	P_i^{ex}	P_i^{in}	PC	PE	MP
PCr	1.00	0.04	-0.01	-0.01	-0.01	0.05	-0.01	0.00	0.00	-0.01	0.00	0.06
ATP	0.04	1.00	0.05	-0.05	-0.01	-0.01	-0.01	-0.01	-0.04	0.02	0.03	0.00
NAD$^+$	-0.01	0.05	1.00	-0.57	-0.36	-0.01	0.02	0.01	0.01	-0.02	0.01	0.03
NADH	-0.01	-0.05	-0.57	1.00	-0.20	0.05	0.01	0.01	0.02	0.00	-0.01	0.01
UDPG	-0.01	-0.01	-0.36	-0.20	1.00	0.00	0.01	-0.03	-0.03	0.03	-0.03	-0.05
GPC	0.05	-0.01	-0.01	0.05	0.00	1.00	0.11	0.05	0.09	0.02	-0.01	0.06
GPE	-0.01	-0.01	0.02	0.01	0.01	0.11	1.00	0.09	0.14	0.04	0.02	0.21
P_i^{ex}	0.00	-0.01	0.01	0.01	-0.03	0.05	0.09	1.00	0.05	0.16	0.21	0.06
P_i^{in}	0.00	-0.04	0.01	0.02	-0.03	0.09	0.14	0.05	1.00	0.13	0.11	0.12
PC	-0.01	0.02	-0.02	0.00	0.03	0.02	0.04	0.16	0.13	1.00	0.15	-0.04
PE	0.00	0.03	0.01	-0.01	-0.03	-0.01	0.02	0.21	0.11	0.15	1.00	-0.12
MP	0.06	0.00	0.03	0.01	-0.05	0.06	0.21	0.06	0.12	-0.04	-0.12	1.00

Metabolites only (LW = 10 Hz)

	PCr	ATP	NAD$^+$	NADH	UDPG	GPC	GPE	P_i^{ex}	P_i^{in}	PC	PE	MP
PCr	1.00	0.02	0.00	-0.01	0.03	0.04	0.02	0.04	-0.01	0.01	0.03	0.01
ATP	0.02	1.00	-0.01	-0.06	0.04	0.00	0.04	0.00	0.03	0.02	0.01	0.04
NAD$^+$	0.00	-0.01	1.00	-0.68	-0.41	0.01	-0.01	0.02	0.01	0.01	0.00	-0.03
NADH	-0.01	-0.06	-0.68	1.00	-0.15	0.00	0.00	-0.02	-0.02	-0.04	-0.01	-0.02
UDPG	0.03	0.04	-0.41	-0.15	1.00	0.01	0.01	-0.02	0.02	0.04	0.01	0.08
GPC	0.04	0.00	0.01	0.00	0.01	1.00	-0.10	-0.01	0.00	-0.02	0.00	-0.27
GPE	0.02	0.04	-0.01	0.00	0.01	-0.10	1.00	0.00	-0.01	-0.03	0.00	-0.03
P_i^{ex}	0.04	0.00	0.02	-0.02	-0.02	-0.01	0.00	1.00	-0.27	-0.05	0.01	0.04
P_i^{in}	-0.01	0.03	0.01	-0.02	0.02	0.00	-0.01	-0.27	1.00	-0.02	0.03	-0.02
PC	0.01	0.02	0.01	-0.04	0.04	-0.02	-0.03	-0.05	-0.02	1.00	-0.24	0.02
PE	0.03	0.01	0.00	-0.01	0.01	0.00	0.00	0.01	0.03	-0.24	1.00	0.03
MP	0.01	0.04	-0.03	-0.02	0.08	-0.27	-0.03	0.04	-0.02	0.02	0.03	1.00

Metabolites + baseline (LW = 10 Hz)

	PCr	ATP	NAD$^+$	NADH	UDPG	GPC	GPE	P_i^{ex}	P_i^{in}	PC	PE	MP
PCr	1.00	0.04	-0.01	0.02	-0.02	0.01	0.02	-0.04	-0.03	-0.04	-0.03	0.12
ATP	0.04	1.00	0.07	-0.07	0.02	0.00	0.00	-0.02	-0.04	0.00	0.01	0.03
NAD$^+$	-0.01	0.07	1.00	-0.65	-0.29	0.02	0.00	0.01	0.05	-0.01	0.03	-0.02
NADH	0.02	-0.07	-0.65	1.00	-0.23	0.00	0.02	-0.02	-0.01	-0.03	-0.03	0.04
UDPG	-0.02	0.02	-0.29	-0.23	1.00	-0.02	-0.05	-0.01	-0.05	0.02	0.00	-0.02
GPC	0.01	0.00	0.02	0.00	-0.02	1.00	0.11	0.12	0.16	0.05	-0.01	0.05
GPE	0.02	0.00	0.00	0.02	-0.05	0.11	1.00	0.23	0.24	0.08	0.03	0.28
P_i^{ex}	-0.04	-0.02	0.01	-0.02	-0.01	0.12	0.23	1.00	0.08	0.20	0.24	0.10
P_i^{in}	-0.03	-0.04	0.05	-0.01	-0.05	0.16	0.24	0.08	1.00	0.20	0.15	0.16
PC	-0.04	0.00	-0.01	-0.03	0.02	0.05	0.08	0.20	0.20	1.00	0.15	-0.06
PE	-0.03	0.01	0.03	-0.03	0.00	-0.01	0.03	0.24	0.15	0.15	1.00	-0.10
MP	0.12	0.03	-0.02	0.04	-0.02	0.05	0.28	0.10	0.16	-0.06	-0.10	1.00

Metabolites only (LW = 20 Hz)

	PCr	ATP	NAD$^+$	NADH	UDPG	GPC	GPE	P_i^{ex}	P_i^{in}	PC	PE	MP
PCr	1.00	0.02	-0.02	0.01	0.04	0.05	-0.02	0.04	0.04	0.00	0.03	-0.03
ATP	0.02	1.00	0.07	-0.13	-0.01	0.02	0.02	0.01	0.01	0.04	0.04	0.03
NAD$^+$	-0.02	0.07	1.00	-0.76	-0.36	-0.03	-0.02	0.00	-0.01	0.00	0.00	0.00
NADH	0.01	-0.13	-0.76	1.00	-0.12	0.06	0.01	0.00	0.01	-0.01	0.00	0.00
UDPG	0.04	-0.01	-0.36	-0.12	1.00	0.01	0.05	0.02	0.03	0.04	-0.02	-0.01
GPC	0.05	0.02	-0.03	0.06	0.01	1.00	-0.17	-0.02	0.01	0.02	0.00	-0.32
GPE	-0.02	0.02	-0.02	0.01	0.05	-0.17	1.00	-0.01	-0.01	0.00	0.03	-0.04
P_i^{ex}	0.04	0.01	0.00	0.00	0.02	-0.02	-0.01	1.00	-0.40	-0.16	0.04	0.01
P_i^{in}	0.04	0.01	-0.01	0.01	0.03	0.01	-0.01	-0.40	1.00	0.01	-0.01	0.00
PC	0.00	0.04	0.00	-0.01	0.04	0.02	0.00	-0.16	0.01	1.00	-0.35	0.02
PE	0.03	0.04	0.00	0.00	-0.02	0.00	0.03	0.04	-0.01	-0.35	1.00	-0.03
MP	-0.03	0.03	0.00	0.00	-0.01	-0.32	-0.04	0.01	0.00	0.02	-0.03	1.00

Metabolites + baseline (LW = 20 Hz)

	PCr	ATP	NAD$^+$	NADH	UDPG	GPC	GPE	P_i^{ex}	P_i^{in}	PC	PE	MP
PCr	1.00	0.08	-0.04	0.02	0.01	0.00	0.05	-0.03	0.01	-0.04	-0.01	0.16
ATP	0.08	1.00	0.06	-0.07	0.05	-0.01	-0.03	-0.03	-0.04	0.02	0.02	-0.01
NAD$^+$	-0.04	0.06	1.00	-0.73	-0.23	0.00	0.00	-0.05	0.02	-0.01	-0.04	0.01
NADH	0.02	-0.07	-0.73	1.00	-0.22	0.01	0.03	0.04	-0.01	0.03	0.02	-0.01
UDPG	0.01	0.05	-0.23	-0.22	1.00	0.00	-0.03	-0.03	-0.04	-0.03	0.02	0.01
GPC	0.00	-0.01	0.00	0.01	0.00	1.00	0.09	0.13	0.19	0.08	-0.01	0.04
GPE	0.05	-0.03	0.00	0.03	-0.03	0.09	1.00	0.20	0.25	0.14	-0.02	0.31
P_i^{ex}	-0.03	-0.03	-0.05	0.04	-0.03	0.13	0.20	1.00	-0.02	0.23	0.22	0.08
P_i^{in}	0.01	-0.04	0.02	-0.01	-0.04	0.19	0.25	-0.02	1.00	0.26	0.13	0.22
PC	-0.04	0.02	-0.01	0.03	-0.03	0.08	0.14	0.23	0.26	1.00	0.07	0.04
PE	-0.01	0.02	-0.04	0.02	0.02	-0.01	-0.02	0.22	0.13	0.07	1.00	-0.11
MP	0.16	-0.01	0.01	-0.01	0.01	0.04	0.31	0.08	0.22	0.04	-0.11	1.00
